# Discourses Delivered before the Cincinnati Medical Library Association

**Published:** 1852-04

**Authors:** 


*Discourses delivered before the Cincinnati Medical Library Association.* By DANIEL DRAKE, M. D., 1852.

Dr. Drake is well known to the medical profession as an industrious, liberal minded, and intellectual man, who has for many years devoted himself with enthusiastic assiduity to the advancement of medicine. Though these discourses have a special local interest, they will be read with pleasure by all who are interested in medicine and medical men.

